# Structures of human PTP1B variants reveal allosteric sites to target for weight loss therapy

**DOI:** 10.1101/2024.08.05.603709

**Published:** 2024-08-07

**Authors:** Aliki Perdikari, Virgil A. Woods, Ali Ebrahim, Katherine Lawler, Rebecca Bounds, Nathanael I. Singh, Tamar (Skaist) Mehlman, Blake T. Riley, Shivani Sharma, Jackson W. Morris, Julia M. Keogh, Elana Henning, Miriam Smith, I. Sadaf Farooqi, Daniel A. Keedy

**Affiliations:** 1 University of Cambridge Metabolic Research Laboratories and NIHR Cambridge Biomedical Research Centre, Institute of Metabolic Science & Addenbrooke’s Hospital; Cambridge, CB2 0QQ, UK.; 2 Structural Biology Initiative, CUNY Advanced Science Research Center; New York, NY 10031, USA.; 3 PhD Program in Biochemistry, CUNY Graduate Center; New York, NY 10016, USA.; 4 PhD Program in Biology, CUNY Graduate Center; New York, NY 10016, USA.; 5 Department of Chemistry and Biochemistry, City College of New York; New York, NY 10031, USA.; 6 PhD Programs in Biochemistry, Biology, and Chemistry, CUNY Graduate Center; New York, NY 10016, USA.

## Abstract

Protein Tyrosine Phosphatase 1B (PTP1B) is a negative regulator of leptin signaling whose disruption protects against diet-induced obesity in mice. We investigated whether structural characterization of human PTP1B variant proteins might reveal precise mechanisms to target for weight loss therapy. We selected 12 rare variants for functional characterization from exomes from 997 people with persistent thinness and 200,000 people from UK Biobank. Seven of 12 variants impaired PTP1B function by increasing leptin-stimulated STAT3 phosphorylation in cells. Using room-temperature X-ray crystallography, hydrogen-deuterium exchange mass spectrometry, and computational modeling, we determined that human variants modulate the 3-dimensional structure of PTP1B through distinct allosteric conduits that energetically link distal, highly ligandable structural regions to the active site. These studies inform the design of allosteric PTP1B inhibitors for the treatment of obesity.

Obesity causes substantial morbidity and mortality due to an increased risk of type 2 diabetes, cardiovascular disease, fatty liver disease, and some cancers (1). A new generation of anti-obesity medications (AOM), the Glucagon-like Peptide-1 (GLP-1) / Gastric inhibitory polypeptide (GIP) / Glucagon receptor agonists, lead to 15–20% weight loss and are transforming the clinical care of people with obesity. However, a significant proportion of people cannot tolerate these medications due to adverse effects, and questions about their suitability for chronic use remain. As such, there is substantial interest in developing new AOM which are safe, effective, and well-tolerated.

Leptin signaling plays a pivotal role in the regulation of appetite and body weight and disruption of leptin or its receptor causes severe obesity in mice and humans. Protein Tyrosine Phosphatase 1B (PTP1B) is a negative regulator of leptin signaling in the hypothalamus, where it dephosphorylates the active site of leptin receptor-associated Janus Kinase 2 (JAK2) and decreases Signal Transducer and Activator of Transcription 3 (STAT3) phosphorylation and transcription of the anorectic neuropeptide, Pro-opiomelanocortin (POMC) (2). Brain-specific (3), leptin receptor-specific (4), and POMC-specific (5) deletion of *Ptp1b* results in mice that exhibit enhanced leptin sensitivity and are protected from high fat diet-induced obesity. As such there has been substantial interest in the development of PTP1B inhibitors for the treatment of obesity. However, this endeavor has proved to be challenging for a number of reasons. Most inhibitors target the catalytic site of PTP1B, but also bind to the highly homologous catalytic site of other PTPs including TCPTP (6), which plays a crucial role in hematopoiesis (7). Compounds that bind effectively to the PTP1B active site tend to be charged, to mimic natural phosphotyrosine substrates, but this property often limits their cell-membrane permeability and bioavailability. Therefore, new approaches to inhibiting PTP1B safely and effectively are needed.

We set out to test whether naturally occurring human variants in PTP1B could be used as tools to identify critical residues and regions of the protein that may be more effectively targeted to develop AOM. To prioritize human PTP1B variants for functional characterization, we studied people with the extreme phenotype of persistent healthy thinness (Body Mass Index, BMI <19 kg/m^2^) recruited into the Study into Lean and Thin Subjects (STILTS cohort; www.stilts.org.uk) (8). Analysis of whole-exome sequencing data on 997 people of UK descent recruited to the STILTS cohort (Fig. 1A, Table S1) identified 29 unrelated people who carried one of six missense variants (allele frequency, AF<1%) in the gene encoding PTP1B (*PTPN1*) of which two people carried a variant which is rare (AF<0.01%) in population cohorts (Q78R, P302Q; Table S2). Among 200,000 unrelated White British exomes from UK Biobank, we identified three predicted protein-truncating PTP1B variants. We also explored whether carriers of any rare missense variants exhibited a trend towards lower or higher mean BMI or proportional BMI categories (BMI >40, BMI >30, BMI <20 kg/m^2^) compared to non-carriers (**Methods**). We selected three additional PTP1B missense variants for exploratory functional characterization: D245G (n=4 carriers; BMI, β (95% confidence interval [CI]) = −5.38 [−9.97, −0.78], glm); L425V (n=4 carriers; β [95% CI] = −4.81 [−9.40,−0.22], glm) and V375M (n=3 of 57 carriers had BMI >40 kg/m^2^; Odds Ratio [95% CI] = 3.1 [0.61–9.4)], Fisher’s exact). Therefore, a total of 12 variants in *PTPN1* encoding PTP1B were taken forward for functional characterization (Fig. 1A, Table S2).

To test whether *PTPN1* variants affect the function of PTP1B protein *in vitro*, HEK293 cells were transiently transfected with constructs encoding wild-type (WT) or mutant PTP1B. WT PTP1B was localized to the endoplasmic reticulum (ER) (Fig. 1B) and suppressed leptin-dependent phosphorylation of STAT3 and transcription of POMC (Fig. S1A). WT PTP1B also decreased basal and BDNF-stimulated phosphorylation of TRKB and insulin-stimulated AKT phosphorylation (Fig. S1A). We investigated whether human PTP1B mutants alter protein expression and/or cellular localization using confocal microscopy of permeabilized transfected cells (Fig. 1B, Fig. S1B–C). Q78R, P302Q, G381S, and A382D significantly increased leptin-stimulated phosphorylation of STAT3; P302Q PTP1B also potentiated POMC transcription, causing a significant loss of function (Fig. 1C–D). Some of these mutants also increased BDNF-stimulated TRKB phosphorylation and enhanced insulin-mediated AKT phosphorylation (Fig. S1D–E). In total, 7 of 12 variants studied caused a statistically significant loss of function (LOF) in one or more assays. Interestingly, V375M PTP1B was mislocalized to the cytoplasm, decreased leptin-stimulated STAT3 phosphorylation to 86% that of WT PTP1B, and decreased POMC transcription, consistent with a significant gain of function (GOF) (Fig. 1B–D, Fig. S1B). P387L PTP1B also caused a decrease in STAT3 phosphorylation compared to WT PTP1B (Fig. 1C,E), consistent with a GOF, although it did not affect POMC transcription.

Many of the PTP1B variants are located in the proline-rich C-terminal region (Fig. 1A). This region is thought to be intrinsically disordered and has not been resolved crystallographically, but regulates PTP1B activity through a variety of mechanisms (9, 10), including binding to SH3 domains (11), intracellular localization to the ER (12), calpain proteolysis (13), and serine phosphorylation (11), that may be disrupted by the mutations. In particular, P302Q is predicted to disrupt the linear LxVP motif (299-LEPP-302) that may be recognized by calcineurin, and P387L is predicted to disrupt the linear SPxK motif (383-QAASPAK-389) that targets Cdc14 and CDKs (14) to S386 for phosphoregulation. In addition, although the C-terminal region of PTP1B is thought to be disordered, NMR experiments indicate that V375 and nearby residues have partial helical character (15). Notably, V375, R373, and R371 are among the residues in PTP1B that experienced the most significant NMR chemical shift perturbations elicited by the allosteric inhibitor MSI-1436 and may form its primary binding site (15). The GOF PTP1B mutation V375M reported here likely exploits the same inherent allosteric wiring in PTP1B to achieve an inverted functional response.

As the disordered C-terminus is challenging to characterize structurally, we sought to investigate the biochemical and biophysical bases of altered PTP1B activity in cells by the clinical variants located in the ordered catalytic domain of PTP1B (Fig. 2A). Several of these mutations are located at or near known allosteric regions. D245G and Q78R are adjacent to the conformationally bistable Loop 16, which is known to be allosterically linked to the bistable, dynamic, catalytically essential WPD loop in the active site (16). Mutational analysis with FoldX (17) suggests that D245G is highly destabilizing (ΔΔG > 4.5 kcal/mol), potentially causing local unfolding. Thus D245G may bias the equilibrium of Loop 16 and allosterically modulate the distal active-site WPD loop.

To study the four mutations in/near the catalytic domain, we performed *in vitro* enzyme activity assays with a purified recombinant construct containing the catalytic domain plus ~20 residues of the disordered C-terminus (residues 1–321; see Methods) (Fig. 2B). D245G and Q78R significantly decreased catalysis, consistent with them having the most extensive LOF effects in cells (Fig. 1). Somewhat surprisingly, I19V also decreased catalysis *in vitro* yet had no statistically significant effects in cells (Fig. 1). These mutations decrease V_max_ but do not change K_m_ (Fig. 2C–D), consistent with allosteric effects from their locations distal from the active site (Fig. 2A).

To attribute more detailed structural mechanism to these catalytic effects, we used room-temperature (RT) X-ray crystallography, which reveals elevated protein conformational heterogeneity that underlies function (16, 18–24). The mutations were evident in 2Fo-Fc and Fo-Fc electron density maps for D245G and I19V (Fig. S2). Difference density for the Q78R mutation was less clear, likely due to this residue’s high surface accessibility and this structure’s relatively lower resolution (Table S4). For D245G in particular, the difference density reveals correlated disappearance of the D245 side chain, appearance of a new ordered water molecule in its place, and perturbation of the neighboring K247 side chain (Fig. 3D). To map detailed, longer-range effects of the mutations on the conformational ensemble of PTP1B, we used integration of absolute difference density above threshold (IADDAT) analysis, as applied previously to time-resolved crystallography (25). With this approach, we observed evidence for conformational disturbances upon mutation that are widespread in the PTP1B structure (Fig. 3). For I19V, IADDAT features span the mutation site and key catalytic loops including the active-site P loop and E loop (24) (Fig. 3A). For D245G, the differences exclude the I19 area, but span an otherwise somewhat similar region of PTP1B, including the D245G mutation site and parts of the active site (Fig. 3C). This pathway also entails the α4 helix, which has been recently shown to influence catalytic activity (26) (see also Fig. 5). For Q78R, the differences are more limited (Fig. 3B), perhaps due to the lower resolution (Table S4). Notably, for each mutant, difference density is minimal at the other mutation sites (gray circles in Fig. 3A–C), suggesting that each mutation affects the conformational landscape of PTP1B in a distinct way.

RT crystallography suggests the mutations have structurally distributed effects on PTP1B in the crystal lattice, but is limited by resolution for some mutant datasets (Q78R) and is restricted to the crystalline environment. To assess whether the mutations also affect the structural dynamics of PTP1B in solution, we used high-resolution local hydrogen-deuterium exchange mass spectrometry (HDX-MS) (27). Local HDX-MS measures the relative exchange of labile amide hydrogens at many overlapping peptide sites in a protein, as a proxy for conformational dynamics (28). Using HDX-MS, we obtained peptide maps of exchange with high (~98.8%) shared coverage of the 1–321 PTP1B sequence across multiple time points for WT and all four mutants in/near the catalytic domain, allowing us to calculate detailed mutant-WT difference Woods plots (Fig. S3–6).

As visualized by peptide “strip” plots, the four mutations have distinct effects on local conformational dynamics throughout the PTP1B catalytic domain (Fig. 4A). Mapping HDX difference values to the protein structure provides further insights into possible allosteric mechanisms underlying the functional effects of the mutations (Fig. 4B–D). For example, I19V decreases exchange in the N-terminal α1’ helical region and many other peptides throughout the protein but has little effect on exchange at the active site (Fig. 4B), consistent with its weaker catalytic effect *in vitro* (Fig. 2) and insignificant functional effects in cells (Fig. 1). By contrast, Q78R decreases exchange for an adjacent buried β-strand and the active-site P-loop, which houses the strictly conserved catalytic cysteine (C215) (Fig. 4C). These regions thus form a conduit from the mutation site to the catalytic center, consistent with significant functional effects for Q78R *in vitro* (Fig. 2) and in cells (Fig. 1). Additionally, D245G increases exchange markedly for the mutation locus itself (Fig. 4D), consistent with FoldX computational predictions and RT crystallography (Fig. 3C–D). It also decreases exchange dramatically for the active-site pTyr recognition loop (Fig. 4D), consistent with D245G exhibiting the most extensive functional impacts *in vitro* (Fig. 2) and in cells (Fig. 1). The allosteric mechanism linking the D245G region to the active site likely entails structural changes on faster timescales than are accessible to HDX-MS experiments or that do not involve backbone amides.

Across the mutations, per-peptide HDX in solution correlates poorly with per-peptide total IADDAT in crystals (Fig. S7). This suggests that crystallographic density and amide hydrogen exchange reveal distinct and complementary aspects of protein conformational heterogeneity and dynamics. This result is consistent with our previous comparisons of HDX-MS for apo WT PTP1B vs. a PTP1B crystal structure pseudo-ensemble (29), which suggested that many protein regions behave differently in these two contexts (27), but expands this view to encompass allosteric mutations.

In contrast to the other catalytic domain mutations, P302Q had no effect on catalysis *in vitro* (Fig. 2A) yet caused a LOF in cells (Fig. 1C–E). P302Q is located just beyond the α7 helix (residues ~284–298) (Fig. 2B), which alternates between ordered and disordered states (16) and has a critical allosteric role in phosphatase function for PTP1B (15, 30) and its close homolog TCPTP (31). Immediately C-terminal to α7, residues 300 and beyond are thought to be intrinsically disordered: for example, P302 is unmodeled in all available crystal structures of PTP1B. However, the AlphaFold 2 (AF2) structural model for full-length PTP1B (32, 33) includes a relatively “confident” prediction that residues 300–303 adopt an ordered conformation near the active-site WPD loop (Fig. 2B). As this conformation conflicts with our crystal form (Fig. S8B), we used local HDX-MS instead to examine P302Q. Many regions positioned near P302 in the AF2 model undergo increased exchange upon P302Q mutation, including the α3 helix and β-sheet lining the allosteric 197 site (16) (Fig. S8A). By contrast, exchange of the catalytic WPD loop and P loop are unaffected, consistent with the lack of catalytic effect for P302Q with purified protein. These results support the AF2 computational model of PTP1B (33). They further suggest that P302Q modulates the PTP1B conformational ensemble in ways that affect function in cells but do not affect inherent catalysis, including potentially altering recruitment of various polypeptide substrates in cells. Such a mechanism would be reminiscent of the auto-regulation of PTP1B’s close homolog TCPTP by its disordered C-terminus (34). Notably, the disordered C-terminus of PTP1B has been successfully targeted with allosteric small-molecule inhibitors (15).

The above analyses focused on characterizing the cellular, biochemical, biophysical, and structural effects of human variants in the enzyme PTP1B. Genetic variants can be used as a tool to inform drug discovery by indicating allosteric weak points on a protein that can be targeted in the drug design process. With this view in mind, we explored hundreds of crystal structures of PTP1B from the Protein Data Bank (PDB) (35), including small-molecule fragment screens (16, 23, 36), to see if ligand binding coincides with mutation sites. Indeed, the pockets near I19V, Q78R, and D245G are bindable by dozens of small-molecule fragments as well as small-molecule buffer components that became fortuitously ordered in crystals (37) (Fig. 5). Two of these fragments allosterically shift the conformation of the WPD loop from open to closed (36); notably, these binding sites are closest to the mutations with the greatest functional impact, Q78R and D245G (Fig. 5). Additionally, in the PTP1B paralog SHP2, the region spanning the residues corresponding to PTP1B D245G and I19V hosts the allosteric small-molecule inhibitor SHP099 (Fig. S9) (38), which stabilizes the interface between the catalytic domain and an SH2 domain to favor an autoinhibitory state. PTP1B lacks SH2 domains, yet the catalytic domains of PTPs are structurally conserved (39), PTPs have been proposed to share common allosteric wiring (40), and much remains unknown about the PTP1B interactome. Taken together, the extensive ligand binding coverage near these three functionally impactful mutations in PTP1B suggests that human mutations can be leveraged to help pinpoint potentially druggable allosteric sites.

Overall, we have studied the human PTP1B protein, which, based on a wealth of preclinical evidence regarding its mechanism of action, is a target for weight loss therapy. Our molecular studies establish that several human variants in *PTP1B* cause a significant LOF and two cause a GOF in cells, providing insights into the molecular mechanisms by which PTP1B structural elements and domains beyond the active site regulate cellular localization and enzymatic function. Several human variants studied here occur at structural locations in PTP1B that have previously been implicated as capable of allostery, both in the ordered catalytic domain (16, 30, 41) and in the intrinsically disordered C-terminal domain (42). Here we have shown that these mutations modulate the conformational ensemble of PTP1B in distinct but overlapping ways and appear to leverage inherent allosteric wiring that energetically links distal, highly ligandable structural regions to the active site. These molecular and structural findings underscore the promise of targeting specific allosteric sites distal to the central catalytic machinery with small-molecule inhibitors for PTP1B (41–44).

Drugs that improve leptin sensitivity (e.g. withaferin) reduce food intake and body weight in obese mice but not in lean mice (45). Clinical trials will therefore be needed to test whether drugs that inhibit PTP1B and thereby increase the amplitude of leptin signaling have a meaningful impact on body weight, either in people with obesity (which may be characterized by a degree of leptin resistance (46)) or in people in the weight-reduced state, where relative leptin deficiency is a major driver of weight regain (47, 48).

## Supplementary Material

Supplement 1

Supplement 2

## Figures and Tables

**Figure 1:**
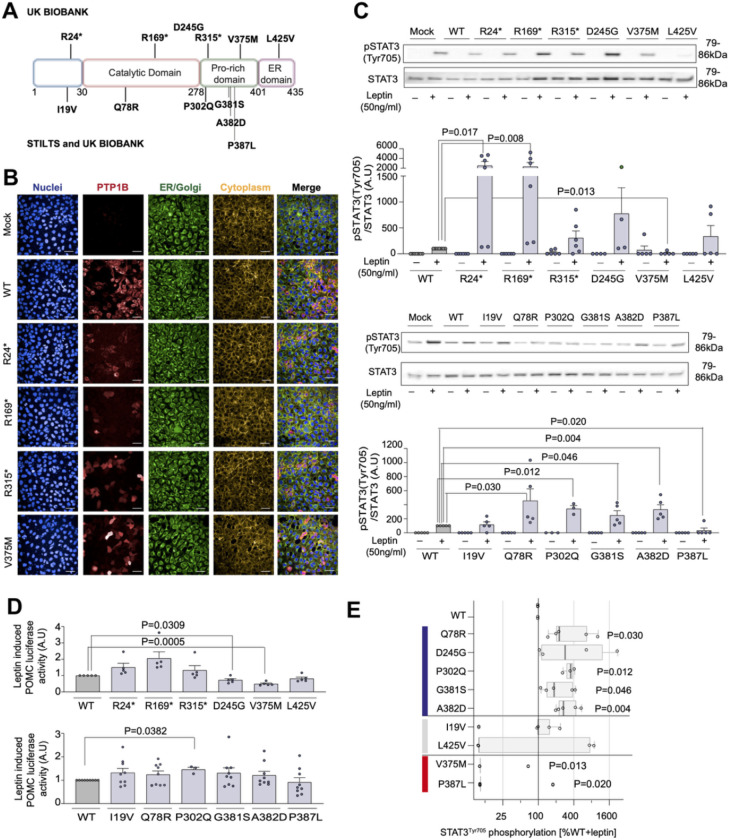
Functional characterization of PTP1B mutants in cells. (**A**) Mapping of variants identified in the STILTS cohort, UK Biobank, or in both cohorts on the structural domains of PTP1B (Proline (Pro)-rich domain; Endoplasmic Reticulum (ER) domain). (**B**) Representative confocal fluorescence microscopy images showing protein localization of WT/mutant PTP1B in HEK293 cells. Blue: DAPI (nuclei), Red: Alexa 647 for HA tagged PTP1B, Green: Alexa 488 for PDI, Yellow: DyLight Phalloidin 554. Scale bar: 50 μm. (**C**) Effect of WT/mutant PTP1B on leptin-stimulated STAT3 phosphorylation (Tyr705). n=4–7; data expressed as mean +/− SEM normalized to WT (0%) and WT leptin-stimulated (100%) (A.U: arbitrary units). Two-tailed unpaired one-sample t-test on log-transformed data for mutant versus WT. (**D**) Effect of WT/mutant PTP1B on leptin induced POMC transcription in a luciferase reporter assay. n= 5–9; data expressed as mean +/− SEM relative to WT. Two-tailed unpaired one-sample t-test for mutant versus WT. (**E**) PTP1B mutations categorized as loss-of-function (LOF; blue), wild-type-like (WT; gray) or gain-of-function (GOF; red) based on phosphorylation and localization assays presented in Fig. 1 and Fig. S1. Statistically significant difference between mutant and WT (expressed as % WT) in leptin-stimulated STAT3 phosphorylation shown. Data are log-transformed; values <10% are set to 10% for visualization. Median shown (box shows interquartile range (IQR); whiskers extend to 1.5*IQR). Results analyzed with an unpaired single-sample t-test (Table S3).

**Figure 2:**
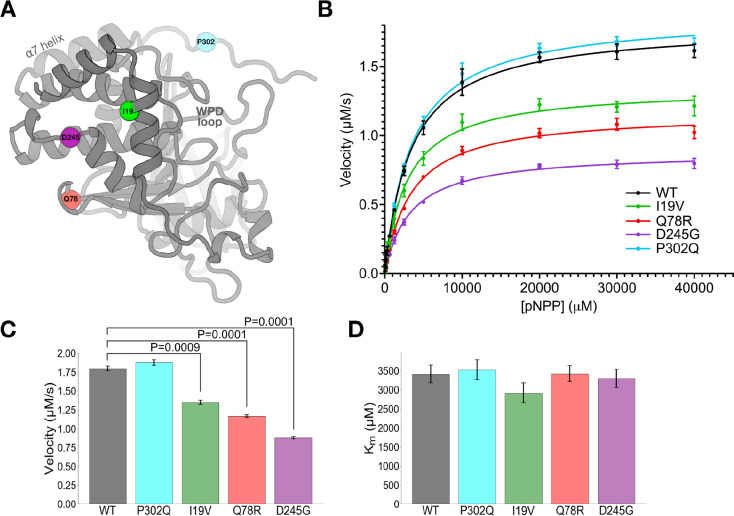
Mutations distal from the active site allosterically perturb enzyme activity *in vitro*. (**A**) Locations of clinically observed mutations in/near the PTP1B catalytic domain mapped to an AlphaFold 2 (32) structural model obtained from the AlphaFold Database (33). Active-site WPD loop and allosteric α7 helix are labeled. (**B**) Michaelis-Menten enzyme activity assays using *para*-nitrophenyl phosphate (pNPP) for mutants vs. WT. Error bars represent 95% confidence intervals. (**C**-**D**) Kinetics parameters from Michaelis-Menten analysis. Error bars represent the bounds of the 95% confidence interval based on 8 replicate results. Statistical significance of mutant vs. WT differences was obtained from two-tailed unpaired one-sample t-tests. (**C**) V_max_ decreases for several mutants, but (**D**) K_m_ is unchanged (all differences between values are not statistically significant at P=0.05).

**Figure 3:**
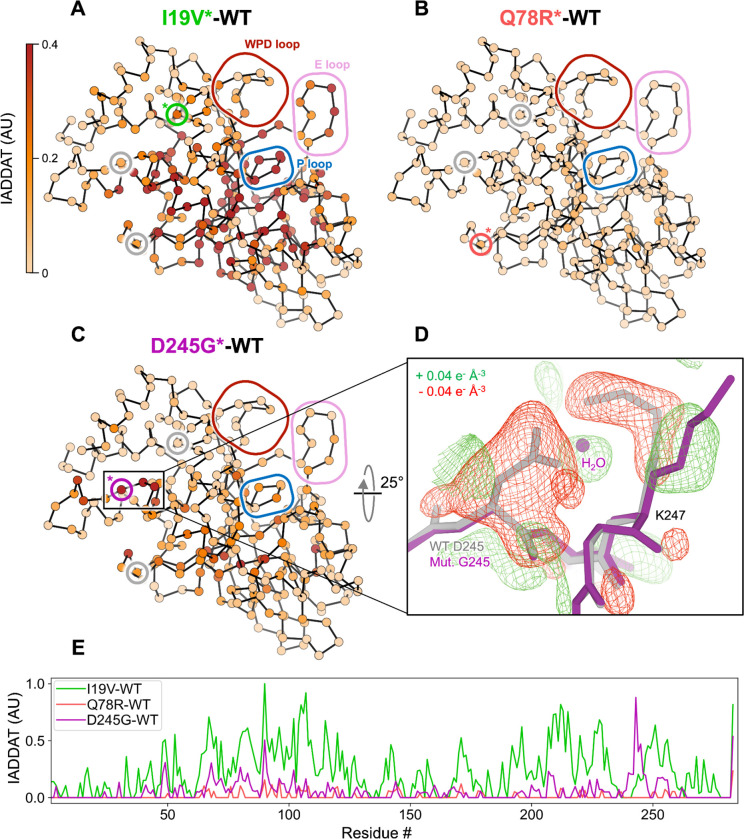
Mutations throughout the catalytic domain alter the conformational ensemble of PTP1B in crystals. (**A**-**C**) Weighted isomorphous difference electron density map (*F*_mut_ − *F*_WT_) features were spatially characterized by integrating the absolute difference density above a noise threshold (IADDAT) (25) for each pair of mutant vs. wild-type PTP1B room-temperature (RT) crystal structures. IADDAT values were averaged per residue and mapped onto the refined RT mutant structures (Cα positions shown as spheres). IADDAT values range from 0 to 1 (arbitrary units, AU) and are capped at 0.4 for visualization. Small colored circles with a colored asterisk highlight the Cα location for each respective mutation (I19V, green; Q78R, red; D245G, purple; circles for other mutations in gray). Large colored rounded boxes highlight key regions of PTP1B. (**A**) I19V shows widespread strong IADDAT, including at several key active-site loops. (**B**) Q78R shows minimal IADDAT, likely due to the lower resolution. (**C**) D245G shows IADDAT for the mutation site (marked with an asterisk inside the inlay box), residues in the α4 helix, and residues in and near the active site. (**D**) Weighted D245G-WT difference electron density (+/− 0.04 e^−^ Å^−3^, green/red), focused on the mutation site, overlaid with wild-type (gray) and D245G mutant (purple) structural models. Surrounding protein and solvent atoms that respond to the mutation are highlighted. (**E**) Plot of per-residue IADDAT vs. sequence for I19V-WT (green), Q78R-WT (red), and D245G-WT (purple).

**Figure 4:**
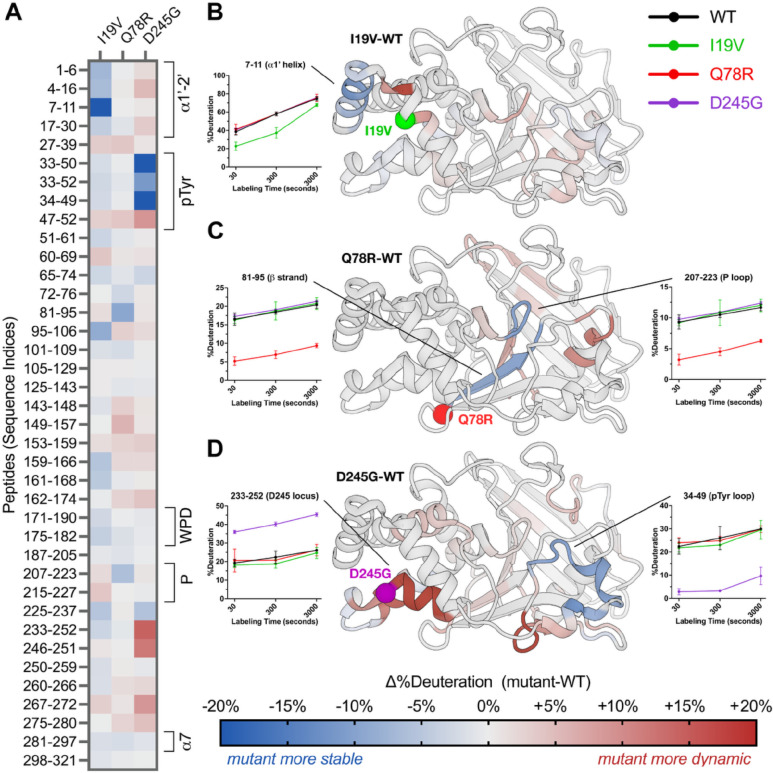
Mutations have widespread effects on protein dynamics in solution by HDX-MS. (**A**) Mutant-WT difference HDX values at 300 seconds of labeling for selected high-quality peptides spanning the PTP1B sequence. Several key structural regions of PTP1B are indicated with brackets. See color bar for corresponding mutant-WT difference HDX values. (**B**-**D**) Mutant-WT difference HDX values at 300 seconds of labeling at the single-amide level (see Methods) mapped to a crystal structure of WT PTP1B (PDB ID: 1T49 (41)) for (**B**) I19V, (**C**) Q78R, and (**D**) D245G. For P302Q, see Fig. S8A. Residues with Δ%deuteration values between −5% and +5% are colored gray for visual clarity.

**Figure 5:**
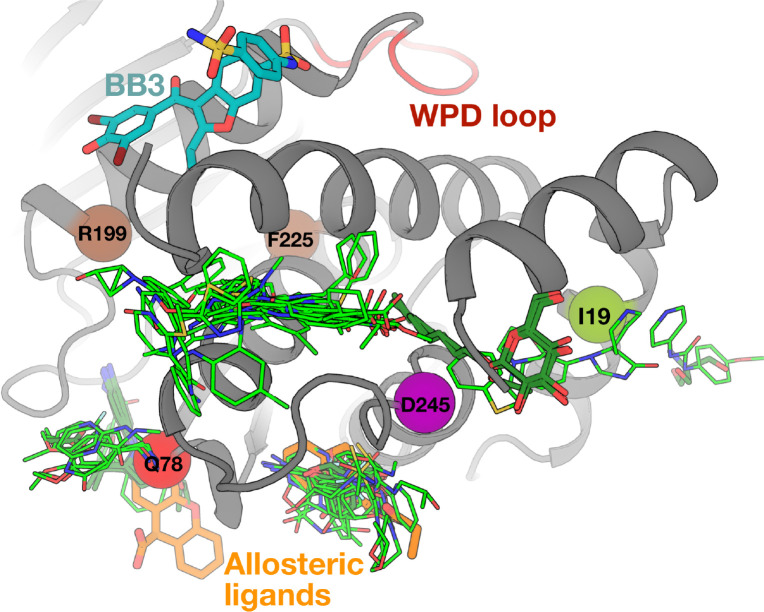
Allosteric sites revealed by human variants are highly ligandable. Ligand binding seen in crystallographic small-molecule fragment screens (light green) (16, 23, 36) and from other structures of PTP1B in the PDB (dark green) indicate significant ligandability at sites near the mutations highlighted in this paper. This includes two fragments (orange, PDB IDs: 7GTT, 7GTV) spanning two sites that were shown to allosterically modulate the active-site WPD loop conformation (top, red) (36). The putative intramolecular network connecting these ligand binding sites to the active site may involve the allosteric BB binding site (cyan) (41) and/or several residues for which mutations were shown to allosterically activate PTP1B activity (brown) (26).

## Data Availability

For X-ray crystallography, the crystal structure coordinates and structure factor data are available at the RCSB Protein Data Bank under the following PDB IDs (accession codes): 9CYO for WT, 9CYP for I19V, 9CYQ for Q78R, and 9CYR for D245G.
