# Supplementary material for: Structures of human PTP1B variants reveal allosteric sites to target for weight loss therapy
Source: bioRxiv. 2024 Aug 7:2024.08.05.603709. Preprint. [Version 1] doi: 10.1101/2024.08.05.603709 (PMC11326154; doi:10.1101/2024.08.05.603709)
Supplement: Supplementary file 1 [file media-1.pdf]

Supplementary Materials for

Structures of human PTP1B variants reveal allosteric sites to target for weight loss therapy

Aliki Perdikari †, Virgil A. Woods †, Ali Ebrahim †, Katherine Lawler, Rebecca Bounds, Nathanael I. Singh, Tamar (Skaist) Mehlman, Blake T. Riley, Shivani Sharma, Jackson W. Morris, Julia M. Keogh, Elana Henning, Miriam Smith, I. Sadaf Farooqi *, Daniel A. Keedy *

† These authors contributed equally to this work.

* Address correspondence to Sadaf Farooqi at isf20@cam.ac.uk and Daniel Keedy at dkeedy@gc.cuny.edu.

The PDF file includes:

Materials and Methods
Figs. S1 to S9
Tables S1, S4

Other Supplementary Materials for this manuscript include the following:

Tables S2, S3, S5

Materials and Methods

Ethics

The study was reviewed and approved by the South Cambridgeshire Research Ethics Committee (12/EE/0172). All participants provided written informed consent prior to inclusion.

STILTS cohort

To recruit people for the Study Into Lean and Thin Subjects (STILTS) cohort we worked in collaboration with 1,143 General Practitioners (GPs) to invite 47,707 eligible people to participate (**Fig. 1A**). Participants of UK European descent, aged 16-65 years and with a BMI <18 kg/m^2 were invited to take part. We applied strict criteria to exclude participants with medical conditions that can affect body weight, such as chronic renal, liver and gastrointestinal problems, or eating disorders assessed using a validated screening tool (SCOFF) (*49*). We also excluded participants who stated that they exercised every day, more than 3 times per week or who reported activity levels more than 6 metabolic equivalents (METs) for any duration or frequency. Finally, we only included participants who reported having been thin their whole life. Some participants with a BMI between 18-19 kg/m^2 were included due to having a strong family history of thinness. One thousand healthy thin participants consented to whole exome sequencing. DNA was extracted from salivary samples obtained using the Oragene 500 kit according to manufacturer's instructions. We obtained additional data on these individuals using the Three Factor Eating Questionnaire which measures cognitive restraint, uncontrolled eating and emotional eating and has been extensively validated in large cohorts (*50*). We also obtained data on occupational and social physical activity using a modified version of the EPIC (European Prospective Investigation into Cancer and Nutrition) physical activity questionnaire, which has been validated against direct measurements of energy expenditure including calorimetry and heart rate variability (*51*).

Variant detection in the STILTS cohort

Exome sequencing was performed for N=997 people from the STILTS cohort. Batch 1 (N=397 people; BGI Genomics) was aligned to hg19 reference (mean depth of target regions, 71-118X (median, 92X); coverage of target regions >= 20X, 70-94% (median, 91%)) and variants were called using GATK v3.3.0 (BGI Genomics). Batch 2 (N=598 people; Macrogen Inc) was aligned to hg38 reference (mean depth of target regions, 102-167X (median, 148X); coverage (%) of target regions with >= 20X, 94-98% (median, 97%) and variants were called using GATK v4.0.5.1 (Macrogen Inc). Variant filtering was based on GATK Best Practices with hard-thresholded variant filters (SNPs: QD<2, FS>60, MQ<40, MRankSum<-12.5, ReadPosRankSum<-8; indels: QD<2, FS>200, ReadPosRankSum<-20). Variant consequence was obtained from Ensembl VEP (Ensembl v96) with respect to PTP1B (gene *PTPN1*) transcript ENST00000371621 and TCPTP (gene *PTPN2*) transcript ENST00000309660. All PTPN1 (PTP1B) and PTPN2 (TCPTP) missense variants from STILTS exomes were taken forward for molecular functional characterization (**Fig. 1A**, **Table S2**).

PTP1B variant selection from UK Biobank 200K exomes

This research was conducted using the UK Biobank Resource (project 53821). The pVCF file for chromosome 20, block 17 was obtained from UK Biobank OQFE 200K exomes interim release (Field 23156). Multiallelics were split and left-normalized. Variants were annotated using Ensembl VEP (v96 cache; GRCh38) and filtered to retain variants with IMPACT=HIGH or MODERATE with respect to PTP1B (gene *PTPN1*) transcript ENST00000371621. Samples were filtered to retain European exomes (Field 22006; self-reported "White British" and tight cluster in genotype PCA). Since kinship was not modeled in this analysis, related pairs up to third-degree kinship were obtained from the UK Biobank Genetic Data resource (ukb_rel.dat) and one person was excluded from each related pair (third-degree kinship). UK Biobank phenotypic and genetic data were obtained for BMI (field 21001.0.0), age, sex, genetic principal components, and WES sequencing batch (50K or 150K).

We identified predicted protein-truncating variants (Ensembl VEP Impact=HIGH, which detected three stop-gain mutations) and, with the aim of augmenting the *PTPN1* missense variants taken forward for exploratory molecular functional characterization, we explored whether any rare missense variants exhibited a trend towards lower or higher BMI, or proportional BMI categories, among carriers compared to non-carriers among unrelated White British exomes from UK Biobank 200,000 exomes. Exploratory single-variant association tests were performed for BMI phenotypes: (i) Fisher's Exact test for dichotomized BMI (BMI >40, BMI >30, BMI <20 kg/m^2) by variant carrier status, (ii) exploratory case-control regression analysis of dichotomized BMI (plink2 –glm with Firth regression) and continuous BMI (plink2 –glm) with covariates age, sex and forty genetic principal components (Fields 22009.0.1-40), together with inspection of the cumulative distribution of BMI and adjusted BMI among variant carriers and non-carriers. Three *PTPN1* stop-gains and three missense variants from UK Biobank 200K exomes were taken forward for exploratory functional characterization (**Table S2**, **Fig. 1A**).

Cell culture

HEK293 cells were cultured in high glucose Dulbecco's modified eagle medium (DMEM, GIBCO, 41965) supplemented with 10% fetal bovine serum (GIBCO, 10270, South America origin), 1% GlutaMAX (100X) (GIBCO, 35050), and 100 units/mL penicillin and 100 µg/mL streptomycin (Sigma-Aldrich, P0781).

Cloning of PTP1B variants for in-cell assays

PTP1B cDNA constructs containing an N-terminal HA tag in pCDNA3.1 (+) vector and TCPTP cDNA constructs containing a C-terminal DYK tag in pCDNA3.1 (+) vector were used throughout the study. Vector containing the N-terminal HA-tagged wild-type (WT) PTP1B (GenScript OHu27552C) and vector containing the C-terminal DYK-tagged wild-type (WT) (GenScript OHu17864D) were used for site-directed mutagenesis using Q5 site-directed

mutagenesis kit (NEB, E0554S) according to the manufacturer's protocols. All constructs were verified with Sanger sequencing.

Cell transfection and stimulation

HEK293 cells were seeded in 96-well plates coated with Poly-D-Lysine (20,000 cells/well). For STAT3 phosphorylation experiments cells were transiently transfected with 100 ng/well plasmid encoding either empty pcDNA3.1(+) vector (negative control), WT or mutant PTP1B or TCPTP plasmid, combined with 50 ng/well plasmid for Leptin Receptor, and 10 ng/well plasmid for STAT3 using Lipofectamine 2000 in Opti-MEM I medium according to the manufacturer's protocols. For TRKB phosphorylation experiments cells were transiently transfected with 100 ng/well plasmid encoding either empty pcDNA3.1(+) vector (negative control), WT or mutant PTP1B plasmid, combined with 25 ng/well plasmid for TRKB Receptor. For AKT phosphorylation experiments cells were transiently transfected with 100 ng/well plasmid encoding either empty pcDNA3.1(+) vector (negative control), WT or mutant PTP1B plasmid using Lipofectamine 2000 in Opti-MEM I medium according to the manufacturer's protocols. After 5 hours transfection, media was replaced by DMEM. Cells were starved overnight, stimulated with either 50 ng/mL leptin (Millipore, 429700) (STAT3 phosphorylation) for 10 minutes, 50 ng/mL BDNF (R&D, 248-BDB-050/CF) (TRKb phosphorylation) for 5 minutes or 200 ng/mL insulin (Sigma, I9278) (AKT phosphorylation) for 10 minutes.

Western blotting

After stimulation cells were washed in PBS and lysed in 40 µL radio-immunoprecipitation assay buffer (RIPA) (Sigma, R0278) supplemented with protease and phosphatase inhibitors while on ice. 96-well plates with cells lysed in the RIPA buffer were shaken vigorously for 30 seconds and kept on ice for 1 minute (x3) until all cells were detached. 30 µL per well of RIPA buffer with lysed cells were transferred to 96 well plates combining 2-4 wells per condition. Plates were kept on ice for 10 minutes and then harvested by centrifugation at 4000 rpm for 20 min and prepared for electrophoresis as described by the manufacturer's protocol using the iBOLT 2 Invitrogen system (B0007, B0009, NW04127BOX, IB23001). Membranes were blocked with 3% BSA solution in TBS-T for 1 hour at room temperature and probed overnight at 4 ºC using Rabbit anti-STAT3 at 1:1000 dilution (Cell Signaling Technology, 12640), Rabbit anti-Phospho STAT3 (pY705) (Cell Signaling Technology, 9145) at 1:1000 dilution, Rabbit anti-Phospho TRKa (pY674/675)/ phospho TRKb (pY706/707) (Cell Signaling Technology, 4621) 1:1000 dilution, Rabbit anti-TRKb (Cell Signaling Technology, 4603) 1:1000 dilution, Rabbit anti-HA (Cell Signaling Technology, C29F4) at 1:1000 dilution, Mouse anti-DYK (FLAG) tag M2 antibody (Sigma, F1804), Rabbit anti-phospho AKT (pS473) (Cell Signaling Technology, 4060) 1:1000 dilution, Rabbit anti-AKT pan (Cell Signaling Technology, 4691) 1:1000 dilution, Rabbit anti-βACTIN (Cell Signaling Technology, 4967) at 1:1000 dilution and Rabbit anti-vinculin (Abcam, ab129002) all prepared in the blocking buffer. Cells were washed three times with TBS-T for 10 min at room temperature with gentle shaking and were incubated with secondary antibody, Goat anti-rabbit IgG-HRP (Dako, P0448) or Goat anti-mouse IgG-HRP (Dako, P0447) diluted 1:2500 in 3% BSA in TBS-T for 1 hour at room temperature. Bands were developed

using the SuperSignal West Dura Extended Duration Substrate (ThermoFisher Scientific, 34075) and imaged in either BioRad Chemidoc XRS or Chemidoc MP Imaging (BioRad) according to the manufacturer's protocols. For total TRKB or AKT, blots were stripped for 15 minutes in 10 mL 1X Re-blot Plus Strong Solution (EMD Millipore, 2504) and blocked with the antibody. The band intensity of western blots was quantified using FIJI. For data normalization in STAT3 or AKT phosphorylation, unstimulated WT PTP1B or TCPTP readouts were set as baseline (0) and maximum WT PTP1B or TCPTP STAT3 or AKT phosphorylation upon stimulation was set as 100%. For data normalization in TRKB phosphorylation, unstimulated WT PTP1B readouts was set as 100%. For data normalization in mock versus WT in STAT3 or AKT phosphorylation unstimulated mock (empty vector) readouts were set as baseline (0%) and maximum mock STAT3 or AKT phosphorylation upon stimulation was set as 100%. For data normalization in mock versus WT in TRKB phosphorylation unstimulated mock (empty vector) readouts was set as 100%. All uncropped blots analyzed are part of Fig. S1.

Luciferase POMC transcription activation assay

HEK293 cells were seeded into white 96-well microplates (chimney well) (Greiner, 655083) coated with Poly-D-Lysine (Sigma, A-003-E) (20,000 cells/well) and transiently transfected the next day with 50 ng/well plasmid encoding either empty pcDNA3.1(+) vector (negative control), WT or mutant PTP1B plasmid, combined with 12.5 ng/well plasmid for Leptin Receptor and 50ng/well plasmid for POMC luciferase using Lipofectamine 2000 (Thermo Fisher Scientific, 11668019) in serum-free Opti-MEM I medium (GIBCO, 31985) according to the manufacturer's protocols. After 5 hours transfection cells were incubated overnight with starvation media (DMEM no FBS) with or without the presence of 200 ng/ml Leptin (Human Recombinant *E. coli*, EMD Millipore, 429700). Quantitation of firefly luciferase activity was performed using the Steadylite Plus Reporter Gene Assay System (Perkin Elmer, 6066759) according to the manufacturer's protocol. For data normalization WT PTP1B POMC luciferase readouts were set to 1 and PTP1B mutant values were normalized relative to WT.

Subcellular localization of human PTP1B mutants

HEK293 cells were seeded in black clear bottom CellCarrier-96 Ultra Microplates (Perkin Elmer, 6055302) coated with Poly-D-Lysine solution (Sigma, A-003-E) (10,000 cells/well) and transiently transfected the next day with 100 ng/well plasmid encoding either empty pcDNA3.1(+) vector (negative control), WT, mutant PTP1B or TCPTP plasmid using Lipofectamine 2000 (Thermo Fisher Scientific, 11668019) in serum-free Opti-MEM I medium (GIBCO, 31985) according to the manufacturer's protocols. After 24 hours, cells were fixed with 4% Formaldehyde (Fisher Chemicals, F/150/PB17) in Phosphate-buffered saline (PBS) for 20 minutes at room temperature, permeabilized with 0.2% Triton X-100 (BDH, 306324N) for 30 minutes at room temperature, blocked for 1 hour in 3% Bovine Serum Albumin (BSA) (Sigma, A7906) in TBS-T at room temperature. For PTP1B localization, cells were incubated overnight at 4°C with Rabbit anti-HA (Cell Signaling Technology, C29F4) at 1:100 dilution in 3% BSA in TBS-T, Mouse anti-PDI (Thermo Fisher Scientific, MA3-018) at 1:100 dilution, or only 3% BSA in TBS-T as a negative control. Cells were washed three times with PBS for 5 minutes, incubated

with goat anti-mouse secondary antibody Alexa Fluor 488 (Thermo Fisher Scientific, A11029) in 1:200 dilution and donkey anti-rabbit secondary antibody Alexa Fluor 647 (Thermo Fisher Scientific, A31573) in 3% BSA in TBS-T for 1 hour at room temperature and washed twice with PBS for 5 minutes, incubated with DAPI (Invitrogen, D1306) in 1:500 dilution in PBS and DyLight 554 Phalloidin (Cell Signaling Technology, 13054) in 1:200 dilution in PBS for 10 minutes, washed once with PBS for 5 minutes and kept in PBS. For TCPTP localization cells were incubated overnight at 4°C with Mouse anti-DYK (FLAG) (Sigma, F1804) at 1:100 dilution in 3% BSA in TBS-T. Cells were washed three times with PBS for 5 minutes, incubated with goat anti mouse secondary antibody Alexa Fluor 488 (Thermo Fisher Scientific, A11029) in 1:200 dilution in 3% BSA in TBS-T for 1 hour at room temperature and washed twice with PBS for 5 minutes, incubated with DAPI (Invitrogen, D1306) in 1:500 dilution in PBS and DyLight 554 Phalloidin (Cell Signaling Technology, 13054) in 1:200 dilution in PBS for 10 minutes, washed once with PBS for 5 minutes and kept in PBS. Cells were imaged in the Opera Phenix High Content Screening Confocal system (Perkin Elmer).

Quantification and statistical analysis of cellular models

Results were analyzed using GraphPad Prism 8 (Graph Pad Software). The difference between mutant PTP1B with WT was estimated and tested using two-tailed one-sample t-tests on original scale or log-transformed data in comparison to either unstimulated or stimulated samples. Nominal $p<0.05$ was considered statistically significant. In **Fig. 1C**, t-tests were run using WT-normalized data on the original scale (**Fig. 1C**, **Fig. S1D-E**, **Table S3**). In **Fig. 1E**, t-tests were run using log(WT-normalized data) with a zero-value offset of 0.001 (**Fig. 1E**, **Table S3**). Missense variants were considered to be loss-of-function (LOF) if the mean of log(WT-normalized data) differed from log(100%) with $p<0.05$ in any of the assays for STAT3, AKT or TRKB phosphorylation upon stimulation (**Fig. 1E**, **Table S3**). Studies in cellular models are from at least 3 independent experiments.

Cloning of PTP1B variants for biophysical experiments

All biophysical experiments were conducted using wild-type PTP1B sequence with residues 1-321 (*16*). The construct is housed in a pET24b vector containing a kanamycin resistance gene. In contrast to some previous related crystallography work with PTP1B, a true WT sequence was used here, with no so-called WT* mutations (C32S/C92V) (*16*). The starting WT construct included amino acid residues 1-435 of PTP1B, then was shortened to residues 1-321 using site-directed mutagenesis. To generate I19V, Q78R, D245G, and P302Q variants, site-directed mutagenesis was employed starting from this 1-321 construct.

The protein expression and purification process followed a previously documented method (*52*), with some slight modifications. To initiate expression, we transformed plasmids containing the desired site mutation into competent *E. coli* BL21 (DE3) cells. These cells were allowed to incubate on LB + kanamycin plates at 37°C overnight. Subsequently, 5 mL starter cultures of LB and kanamycin at 1 mM final concentration were inoculated with individual colonies and shaken overnight at 37°C. Larger 1 L cultures of LB and kanamycin at 1 mM final concentration were

then inoculated with starter cultures and grown with shaking at 37°C until the optical density at 600 nm reached approximately 0.6–0.8. Induction of the 1 L cultures was carried out by IPTG at a final concentration of 500 µM, and shaking was continued overnight at 18°C. Induced cells were harvested by centrifugation, the resulting cell pellets ("cellets") were flash frozen, and stored at -80°C in 50 mL conical tubes, for subsequent purification.

Prior to purification, harvested cells were resuspended with lysis buffer containing dissolved Pierce protease inhibitor tablets using a vortexer. Resuspended cells were sonicated (on ice) for 10 mins at an amplitude of 50% using 10 s on/off intervals. Cells were then centrifuged and the supernatant syringed filtered with a 0.22 µm filter and reserved for purification. Initial purification involved cation exchange on an SP FF 16/10 HiPrep column (GE Healthcare Life Sciences). This was carried out using a lysis buffer (100 mM MES pH 6.5, 1 mM EDTA, 1 mM DTT) and a NaCl gradient (0-200 mM), resulting in protein elution occurring at approximately 200 mM NaCl. Following this step, size exclusion chromatography was conducted utilizing a S75 size exclusion column (GE Healthcare Life Sciences) in a crystallization buffer (10 mM Tris pH 7.5, 0.2 mM EDTA, 25 mM NaCl, 3 mM DTT). The purity of the sample was evaluated through SDS-PAGE analysis, demonstrating it to be devoid of contaminants and displaying a high level of purity.

In vitro enzyme activity assays

To investigate the kinetic parameters of the mutant proteins, a colorimetric assay utilizing *para*-nitrophenyl phosphate (pNPP) as a substrate was executed. The assay buffer was prepared to a final concentration of 50 mM HEPES (pH 7.0), 1 mM EDTA, 100 mM NaCl, 0.05% Tween-20, and 1 mM β-mercaptoethanol (BME), then 0.22 µm filtered and stored at room temperature.

Subsequently, a series of 12 pNPP concentrations, ranging from 40 mM to 3.9 µM, were prepared by serial dilution in the assay buffer to cover a broad range of substrate concentrations for kinetic analysis. Prior to the assay, two independent measurements of each mutant protein's concentration were performed five times using a NanoDrop One. Afterwards, the mutant protein samples were diluted to matching concentration (250 nM) in assay buffer, and the average protein concentration for each mutant protein was confirmed again.

For the assay, 50 µL of the diluted protein solution was aliquoted into each well of a Corning 96-well flat-bottom non-binding polystyrene plate. The reaction was initiated by adding 50 µL of the pNPP solution to each well, with thorough mixing achieved through gentle pipetting. Absorbance at 405 nm was measured at 18-second intervals over a 6-minute period using a SpectraMax i3 plate reader, calibrated prior to use.

Each concentration of pNPP was tested in quadruplicate for each mutant protein. The slopes of absorbance change (mAU per minute) over the 6-minute interval were calculated and used to derive the maximum velocity (V_{max}) of each reaction. The catalytic constant (k_{cat}) was determined by dividing V_{max} by the average concentration of each mutant protein. These k_{cat} values, obtained from two independent experiments, were combined, and analyzed using GraphPad

Prism 9 to plot the kinetic curves and to calculate the Michaelis constant (K_m), with results presented in **Fig. 2**.

Protein crystallization

Purified WT PTP1B mutants were concentrated to a final concentration of about 40 mg/mL before initiating crystallization experiments. The crystallization well solution used in this study consisted of 0.3 M magnesium acetate, 0.1 M HEPES pH 7.5, 0.1% β-mercaptoethanol, 13.5% PEG 8000, 2% ethanol. Crystallization drops were prepared using EasyXtal 15-Well trays (Nextal, 132006). A 1:1 protein-to-solution ratio was employed such that 1 μL of protein solution was mixed with 1 μL of well solution for one set of drops, and 2 μL of protein solution was mixed with 2 μL of well solution for another set of drops. The prepared crystallization trays were then incubated at a controlled temperature of 4℃. Within a short span of approximately 2 days, crystals became evident in the trays. These initial crystals progressively grew over the course of about 1 week, reaching their maximum size during this period. The fully developed crystals obtained final dimensions ranging from approximately 50 x 50 x 100 μm to 300 x 300 x 1000 μm. I19V crystals were generally largest, and Q78R crystals generally the smallest.

X-ray diffraction

Crystals for room-temperature (RT) X-ray data collection were harvested under a humidifier using MiTeGen microloops of relevant size and stored in SSRL *In-Situ* Crystallization Plates, allowing crystals to be shipped in humidity controlled chambers for remote data collection at elevated temperature. X-ray diffraction data were collected remotely at the Stanford Synchrotron Radiation Lightsource (SSRL) beamline 12-1. Single crystals were exposed to X-rays at an elevated cryostream temperature of 298 K, with 0.5° of helical crystal translation per image and 0.1 seconds exposure per image, for a total of 180° across 360 images. The unattenuated X-ray flux was measured as 2.0×10^{12} photons/second, and the X-ray beam attenuated to between 1-5% transmission.

Crystallographic data reduction and refinement

The data reduction pipeline fast_dp (*53*) was used for initial bulk data reduction in order to determine the highest-resolution datasets to process. Selected datasets were reprocessed using the xia2 DIALS (*54*) pipeline. Ideal resolution cutoffs were automatically determined based on a $CC_{1/2}$ (*55*) of ~0.3 and an outer shell completeness of >97%. Molecular replacement and initial refinement were performed using MOLREP and Refmac5 from the CCP4 suite of crystallographic programs (*56*), using PDB entry 1SUG as the initial molecular replacement search model. Subsequent iterative rounds of refinement were performed using phenix.refine (*57*) and Coot (*58*). Hydrogen atoms were added using phenix.ready_set (*59*, *60*). Final PTP1B models were assessed statistically using MolProbity (*61*). Data collection and refinement statistics are shown in **Table S4**.

Density map analysis

In order to reduce the noise in our difference electron density maps, we reweighted our maps using the following method. PTP1B mutant unmerged data from DIALS were run through the data reduction program Aimless (*62*) in order to rescale the data to a reference dataset. In this case, PTP1B WT experimental data (structure factor amplitudes only) were utilized as reference data to solve indexing ambiguity and space group, and to provide a free reflections set for calculating R_{free}. Fo-Fo difference structure factors were then weighted using custom scripts within the reciprocalspaceship suite (*63*), with map phases calculated using a PTP1B WT model refined against structure factor amplitudes (*F*) only. Structure factors were then converted to difference maps using reciprocalspaceship using weights with an alpha value of $\alpha = 0.05$ to decrease the influence of measurement errors on structure factor difference amplitudes.

To characterize our difference electron density map features across the structure of our PTP1B mutants, we integrated the absolute difference density above a noise threshold (IADDAT) (*25*). Here, difference peaks with an absolute value greater than 0.04 e^{-} $Å^{-3}$ (converted from equivalent RMSD values in Coot) and within 2.5 Å of a protein heavy atom (excluding waters) were summed and averaged on a per-residue basis. These values were subsequently mapped back onto the corresponding Cα positions in the protein crystal structure. This was achieved using custom scripts within reciprocalspaceship. Jupyter notebooks for structure factor reweighting and IADDAT calculations are available at GitHub repositories associated with reciprocalspaceship (*63*) and Wolff et al. (*25*) respectively.

To compare crystallographic IADDAT values to HDX-MS data (see below) for the peptides in **Fig. S7**, we calculated the IADDAT average of the reportable residues for each peptide in the digest.

HDX-MS data collection

Sample Handling
HDX-MS data collection and processing were performed as recently reported (*27*) with some minor variations. For each experiment, liquid handling was performed by the LEAP HDX platform. This robotic system precisely initiates and times labeling reactions (reaction volume of 50 µL), followed by rapid mixing with quench solution and dropping the temperature to 0-4°C. Once the sample was thoroughly mixed with quench, 100 µL of this quenched sample was then injected into the pepsin column.

Digestion Optimization
Digestion optimization experiments were conducted to determine the optimal quench conditions to halt hydrogen-deuterium exchange and prepare the protein for digestion over a pepsin column by inducing partial or extensive denaturing / unfolding. A series of quenching solutions consisting of 1.5% formic acid, 3.0% acetonitrile, and varying concentrations (0, 0.5, 1.0, 2.0, 4.0 M) of guanidinium hydrochloride (GuHCl) was mixed in a 1:1 ratio with the unlabeled protein. The deuteration buffer was identical to the protein buffer used in the non-deuterated experiments, except for the presence of deuterium in high (>99.5%) abundance. To perform

local hydrogen-deuterium exchange (HDX) experiments, a Waters Enzymate BEH Pepsin Column was employed to generate peptides for subsequent analysis. Peptic peptides were eluted through a C18 analytical column (Hypersil Gold, 50 mm length × 1 mm diameter, 1.9 μm particle size, Thermo Fisher Scientific) into a Bruker maXis-II ESI-QqTOF high-resolution mass spectrometer. Peptide maps were generated for PTP1B for each of the varied GuHCl conditions. The best coverage and highest resolution were seen with conditions 2.0 M and 4.0 M. A concentration of 3.0 M of GuHCl was chosen for future experiments.

HDX Labeling

Purified protein samples (PTP1B 1-321 WT, I19V, Q78R, D245G, P302Q) were prepared as described in the previous section. All experiments were carried out in the crystallization buffer at 15°C to best match pH, salt, and reducing conditions present in comparable structural studies of PTP1B. The protein sample was first diluted to 20 μM in the H_2O crystallization buffer. The protein was then mixed with the labeling (D_2O) buffer, whose chemical composition was identical to the H_2O crystallization buffer. The labeling reaction mixture consisted of 1 part protein (20 μM in H_2O crystal buffer) and 9 parts D_2O crystal buffer for a total D_2O content of 90% for the reaction duration. The reaction was quenched after timepoints of 30 s, 100 s, 300 s, 1000 s, 3000 s, and 10,000 s. In order to stop the HDX labeling process, a cold quenching solution (1.5% formic acid, 3.0% acetonitrile, and 3 M GuHCl) was mixed in a 1:1 ratio with the labeled sample. The LEAP HDX system injected 100 μL of this solution into a pepsin column for digestion and further analysis. MS/MS fragmentation was used to confirm peptide identity along the sequence of PTP1B 1-321 WT, and these peptides and their retention times were used by the HDExaminer software to identify them automatically in the 'on-exchange' experiments. In order to correct for back exchange in the regular and quantitative experiments, fully deuterated (FD) preparations were carried out on each PTP1B variant (PTP1B 1-321 WT, I19V, Q78R, D245G, P302Q). The protein was incubated at room temperature (20°C) for 28 days in a 90% D_2O/H_2O crystallization buffer. Duplicate (n=2) experiments were run for the 30 s and 300 s time points, while the 3000 s results are based on a single experiment in the WT, I19V, Q78R, and D245G variants.

<u>HDX-MS data analysis</u>

Data Preprocessing

Before performing the HDX analysis, the raw mass spectrometry data files (.d format) were processed using Compass Data Analysis 5.3 and Biotools 3.2 software to convert the data into a suitable format (.csv). The preprocessed data files were imported into version 3.3 of the HDExaminer software from Sierra Analytics. The imported data files were aligned based on the peptide identification information, retention time, and m/z values for accurate analysis. This step ensures that the corresponding peptide measurements from different time points are properly aligned for further analysis. The imported data were matched with the peptide sequences derived from the pepsin-based protein digestion within HDExaminer.

Peptide-Level Exchange Rate Analysis

The deuteration level of each peptide was determined by comparing the centroid mass of the deuterated peptide ion with that of the corresponding non-deuterated peptide ion at each time point. This calculation provides the deuteration percentage for each peptide at different time intervals. The HDExaminer software employs various algorithms to calculate the exchange rates of individual peptides. These algorithms utilize mathematical models, such as exponential fitting, to estimate the exchange kinetics and determine the exchange rates of the identified peptides. Relative deuterium uptake is expressed as the peptide mass increase divided by the number of peptide backbone amides. When the second residue of the peptide is not proline, the number of peptide backbone amides was decreased by one to account for rapid back-exchange by the amide adjacent to the N-terminal residue.

Visualization and Data Interpretation

The exchange rate profiles at the peptide and residue levels were computed in HDExaminer. The software provides graphical representations, such as heat maps and exchange rate plots, to facilitate the interpretation of the data. The exchange rate profiles can be further analyzed to identify protein regions exhibiting differential exchange behavior under different experimental conditions. To qualitatively visualize exchange values mapped to 3D protein structures, we obtained estimates of deconvoluted and smoothed residue-level interpolation of the peptide results from HDExaminer and plotted values along color spectra for Δ%deuteration (Fig. 4) that were then mapped onto the structures for the five protein variants (PTP1B 1-321 WT, I19V, Q78R, D245G, P302Q).

<u>Structural modeling and visualization</u>

The structural models for full-length PTP1B predicted by AlphaFold 2 (*32*) were obtained from the AlphaFold database (*33*); these models include the catalytic domains plus the mostly disordered C-termini, which have not been structurally resolved experimentally, with varying annotated degrees of confidence for different residues. The sites of human variant mutations were mapped to the structures and visualized in the context of different ligands and crystal lattice contacts using PyMol (*64*). Predictions of the effect of point mutations on structural stability were performed using FoldX v5 (*17*). Final structural visualization and image production was performed using PyMol.

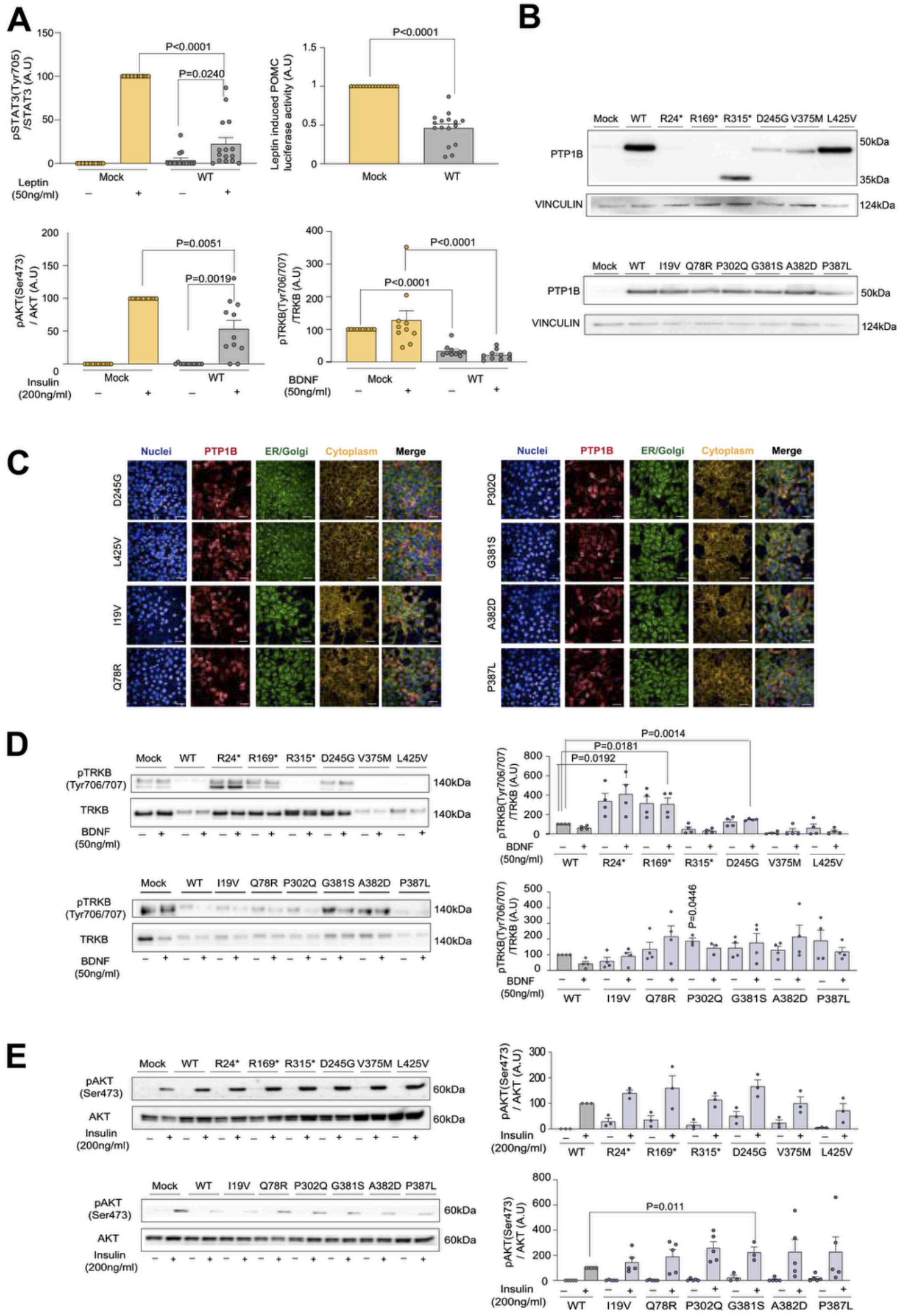

Figure S1: Functional characterization of WT and mutant forms of PTP1B.
(**A**) Effect of overexpression of human WT PTP1B in HEK293 cells on leptin-stimulated STAT3 phosphorylation (Tyr705) and POMC luciferase reporter activity, insulin-stimulated AKT phosphorylation

(Ser473) and BDNF-stimulated TRKB phosphorylation (Tyr706/707) compared to mock-transfected cells. n= 10-17; mean +/- SEM, normalized to mock (0%) and mock stimulated (100%) (A.U: arbitrary units). Two-tailed unpaired one-sample t-test for WT PTP1B versus mock.

(**B**) Western blots depicting expression of WT/ mutant PTP1B in HEK293 cells, n=1.

(**C**) Representative confocal fluorescence microscopy images showing protein localization of WT/mutant PTP1B in HEK293 cells. Blue: DAPI for nuclei, Red: Alexa 647 for HA tagged PTP1B, Green: Alexa 488 for PDI, Yellow: DyLight Phalloidin 554. Scale bar: 50 µm.

(**D**) Effect of WT/mutant PTP1B on BDNF-stimulated TRKB phosphorylation. n=3-4; data are expressed as mean +/- SEM normalized to WT (100%) (A.U: arbitrary units). Two-tailed unpaired one-sample t-test on log-transformed data for mutant versus WT.

(**E**) Effect of WT/mutant PTP1B on insulin-stimulated AKT phosphorylation. n=3-5; data expressed as mean +/- SEM, normalized to WT (0%) and WT stimulated (100%) (A.U: arbitrary units). Two-tailed unpaired one-sample t-test on log-transformed data for mutant versus WT.

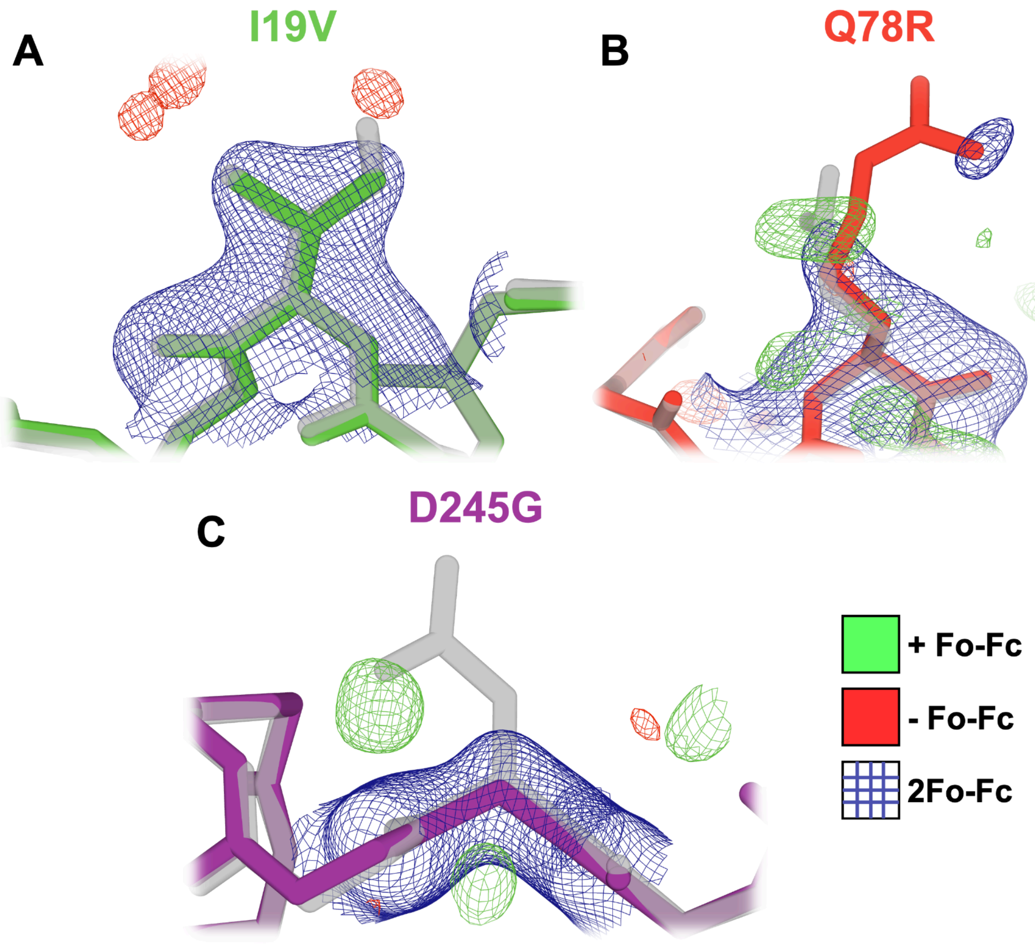

Figure S2: Crystallographic electron density for mutations.
Mutant crystal structure 2Fo-Fc and Fo-Fc electron density maps contoured at 1 σ for 2Fo-Fc (blue mesh) and +/- 3 σ for Fo-Fc (green/red mesh).
(**A**) The I19V mutant crystal structure (green sticks) presents no apparent 2Fo-Fc electron density for the delta carbon present in wild-type isoleucine (gray transparent sticks).
(**B**) The Q78R mutation site in its respective crystal structure (red sticks) is less clear. This is likely a function of the relatively poor resolution compared to the other mutant structures (2.30 Å in Q78R vs. 1.99 Å in I19V and 1.65 Å in D245G). Further, the exposure of the residue to solvent channels in the crystal makes it more likely for the side chain to adopt multiple conformations, and therefore challenging to resolve in electron density maps at low relative resolution.
(**C**) 2Fo-Fc electron density at the D245G mutation site (purple sticks) shows clearly that the entire wild-type aspartic acid side chain is absent from the D245G mutant crystal structure.

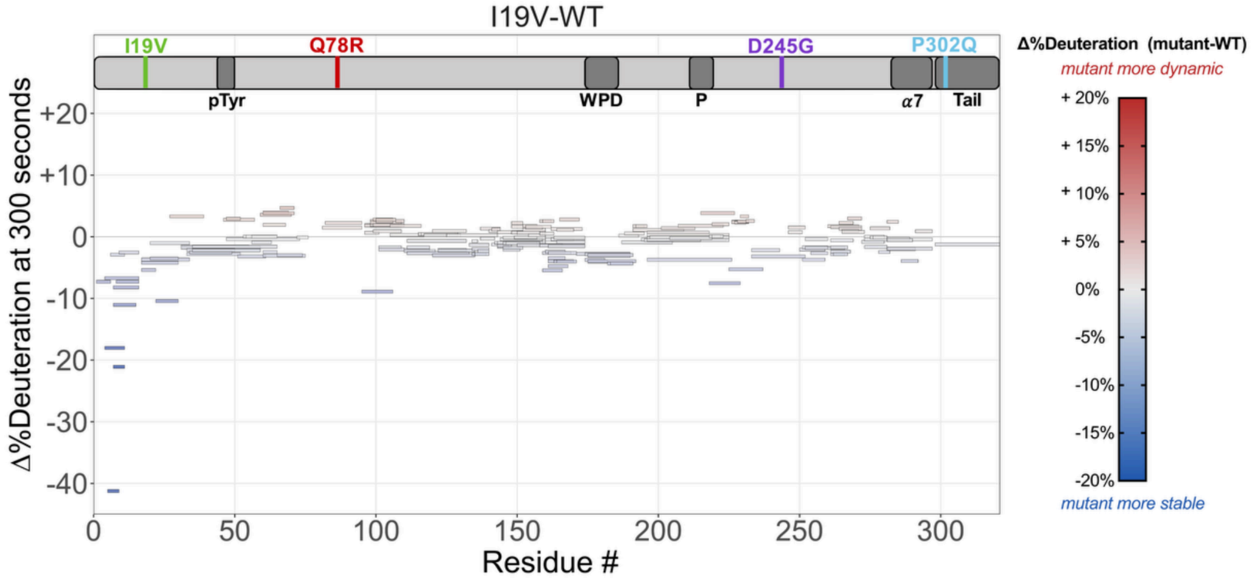

Figure S3: HDX-MS difference Woods plots of I19V.
The difference in %deuteration values at 300 seconds for the peptides of the I19V mutant PTP1B minus the values for WT PTP1B, plotted against amino acid sequence.

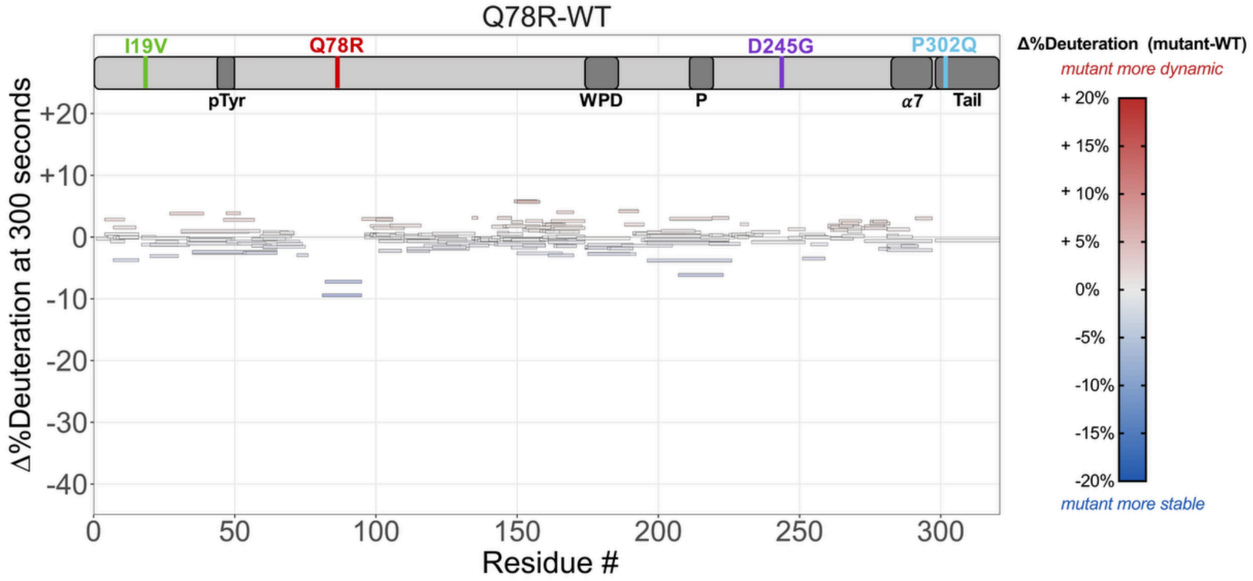

Figure S4: HDX-MS difference Woods plots of Q78R.
The difference in %deuteration values at 300 seconds for the peptides of the Q78R mutant PTP1B minus the values for WT PTP1B, plotted against amino acid sequence.

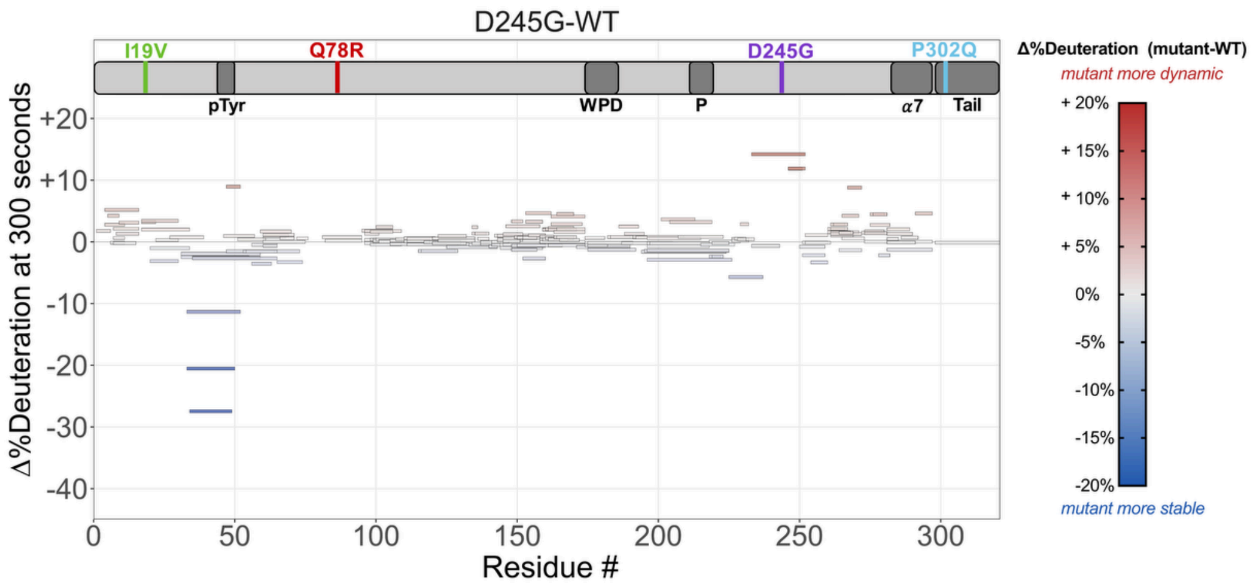

Figure S5: HDX-MS difference Woods plots of D245G.
The difference in %deuteration values at 300 seconds for the peptides of the D245G mutant PTP1B minus the values for WT PTP1B, plotted against amino acid sequence.

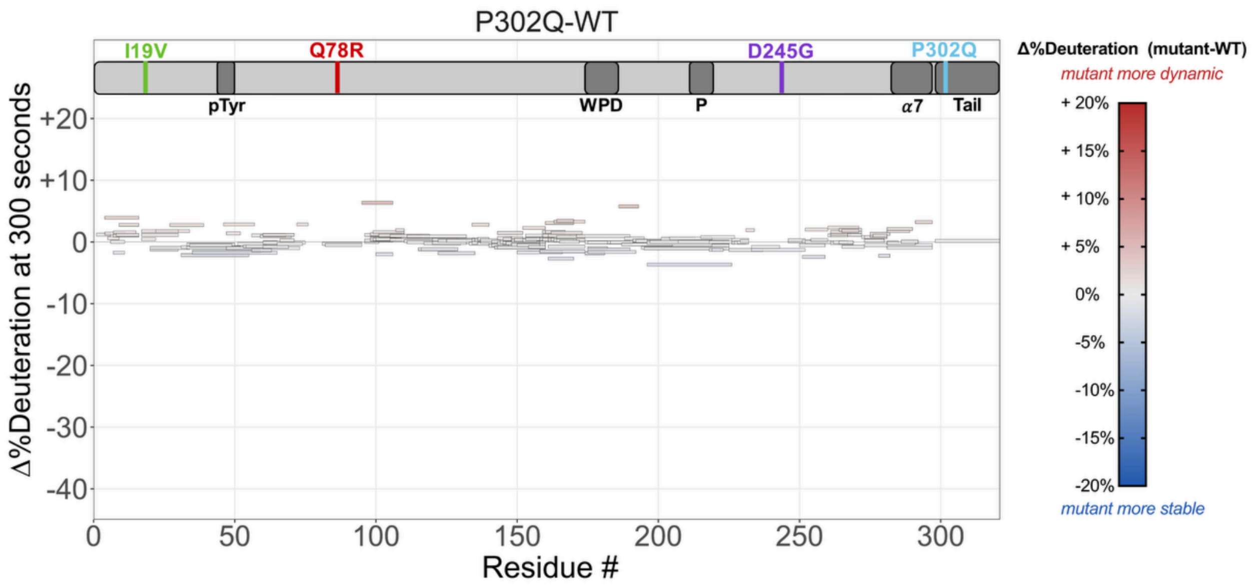

Figure S6: HDX-MS difference Woods plots of P302Q.
The difference in %deuteration values at 300 seconds for the peptides of the P302Q mutant PTP1B minus the values for WT PTP1B, plotted against amino acid sequence.

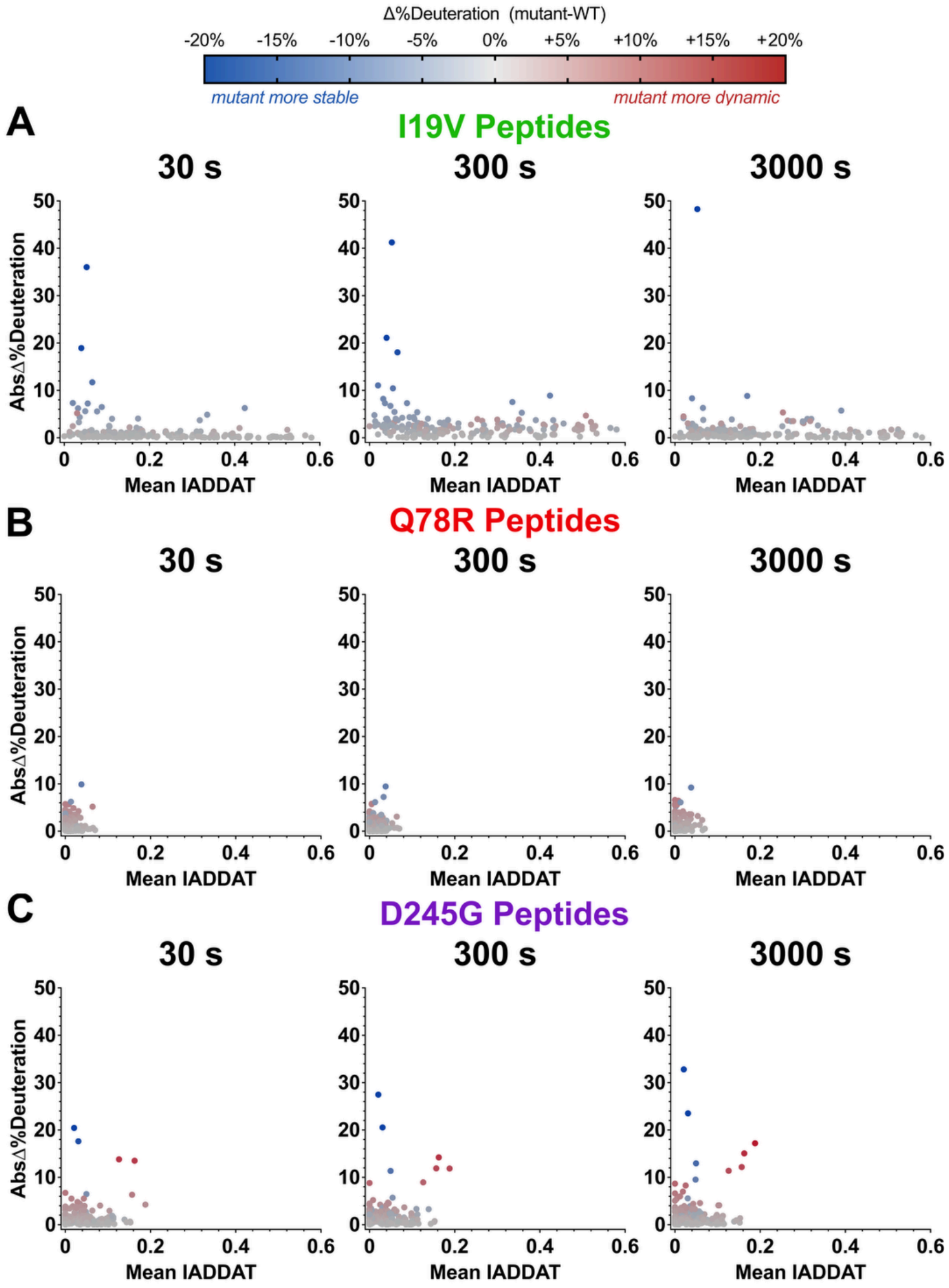

Figure S7: Mutations affect distinct structural regions in crystal vs. in solution.
Absolute values of change in HDX-MS of peptides in the mutants over WT PTP1B at different labeling times vs. crystallographic absolute difference electron density above a noise threshold (IADDAT) values averaged for each reportable residue in the peptide. The first residue of each peptide is subject to rapid back-exchange during LC/MS and is excluded from the IADDAT average. Colors indicate the direction of Δ%deuteration for each peptide: decrease for the mutant (blue), no change (gray), and increase for the mutant (red). (**A**) I19V, (**B**) Q78R, (**C**) D245G.

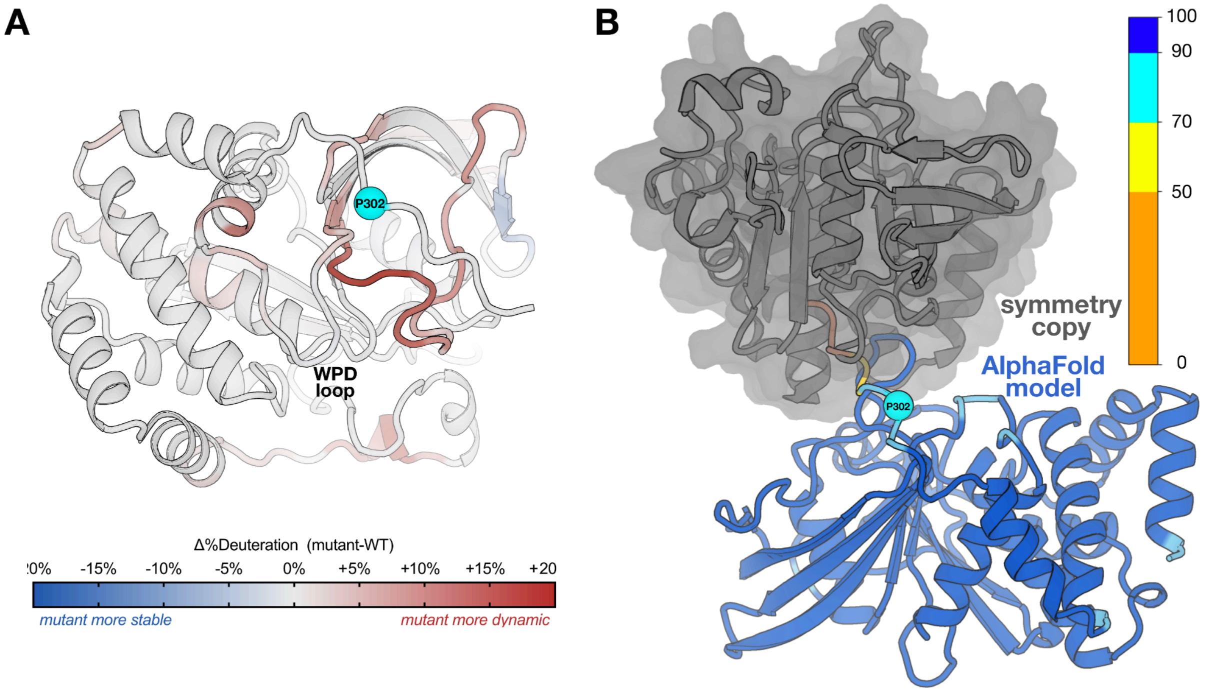

Figure S8: Dynamic effects from a predicted quasi-ordered P302Q conformation.
(**A**) HDX-MS results for P302Q-WT mapped onto a structural model obtained from AlphaFold DB (*32*, *33*) only showing residues 1-310 for simplicity and to include the predicted location of the P302Q mutation. See color bar for corresponding mutant-WT difference HDX values at 300 seconds of labeling. Residues with Δ%deuteration between -5% and +5% are colored gray for visual clarity.
(**B**) The AlphaFold Database model (*32*, *33*) of full-length PTP1B (residues 1-310 shown) colored according to the AlphaFold 2 predicted local distance difference test (pLDDT), along with an aligned symmetry mate from our $P3_121$ crystal form (gray), together show how crystal contacts impede the ability to resolve the predicted conformation of the C-terminal region including P302 in crystal structures.

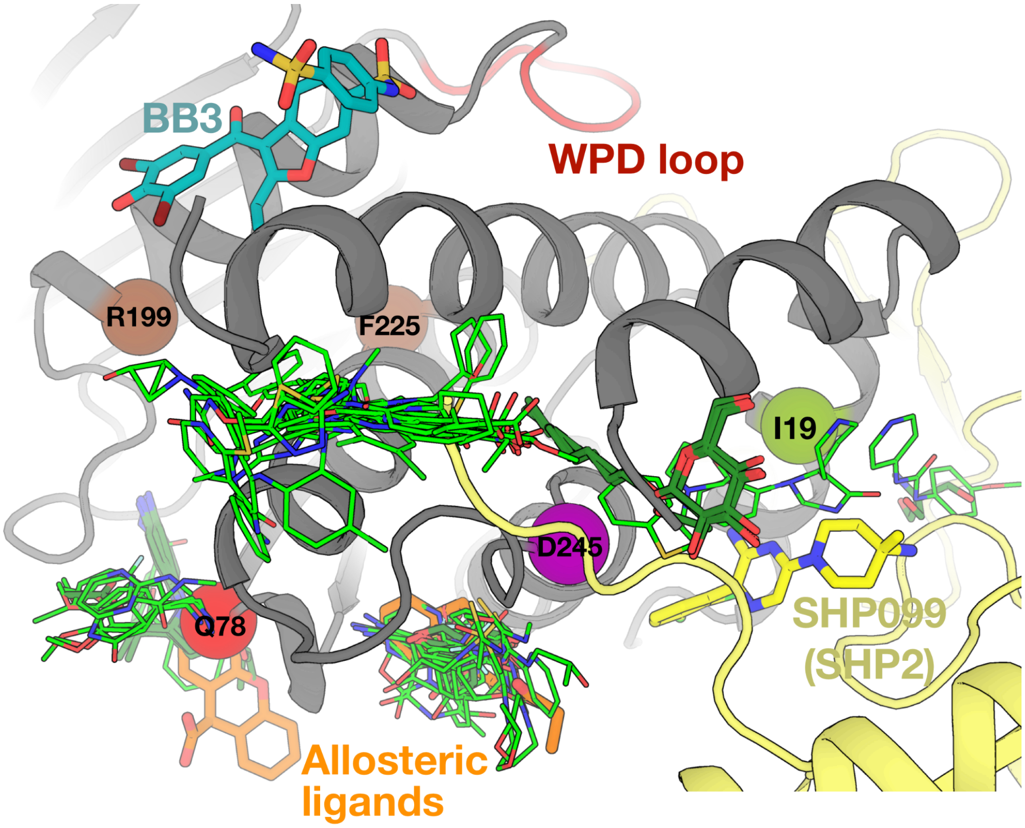

Figure S9: SHP2 allosteric modulator binds near mutation sites.
Same as **Fig. 5** with the addition of the allosteric modulator of the PTP1B homolog SHP2, SHP099 (yellow), and the SH2 domain of SHP2 (pale yellow) (PDB ID: 5EHR) (*38*).

	Sequencing provider		
	**BGI**	**Macrogen**	**Combined**
**Number of participants sequenced**	399	598	997
**Number of males (%)**	64 (16)	111 (18.6)	175 (17.6)
**Number of females (%)**	335 (84)	487 (81.4)	822 (82.4)
**Average age in years (SD)**	43.2 (14.6)	36.7 (13.5)	39.3 (14.4)
**Average BMI in kg/m^2 (SD)**	17.6 (0.93)	17.5 (0.89)	17.5 (0.91)
**Always thin (%)**	356 (89.2)	575 (96.2)	931 (93.4)
**Family history of thinness (%)**	301 (75.4)	521 (87.1)	822 (82.4)

Table S1: STILTS cohort participants who underwent whole exome sequencing.

Table S2 is provided as an XLSX file.

Table S2: PTP1B and TCPTP variants selected for molecular functional characterization from STILTS exomes and UK Biobank 200,000 exomes.

Table S3 is provided as an XLSX file.

Table S3: Statistical analysis of molecular assays for the characterization of PTP1B variants.

**PDB ID**	*9CYO*	*9CYP*	*9CYQ*	*9CYR*
**Construct**	WT	I19V	Q78R	D245G
**Temperature (K)**	298 K	298 K	298 K	298 K
**Resolution (Å)**	43.82-1.94 (2.01-1.94)	44.76-1.99 (2.06-1.99)	43.86-2.30 (2.39-2.30)	38.79-1.65 (1.71-1.65)
**Completeness (%)**	99.80 (99.92)	99.78 (100)	97.99 (98.40)	99.88 (99.16)
**Multiplicity**	9.7 (9.8)	9.1 (9.0)	10.2 (10.7)	9.7 (7.2)
**I/sigma(I)**	8.96 (0.83)	6.02 (0.91)	5.37 (0.37)	16.67 (1.02)
**R_{merge}(I)**	0.143 (2.884)	0.187 (2.611)	0.276 (3.299)	0.053 (1.719)
**R_{meas}(I)**	0.151 (3.043)	0.198 (2.771)	0.291 (3.466)	0.056 (1.853)
**R_{pim}(I)**	0.048 (0.961)	0.065 (0.921)	0.090 (1.055)	0.018 (0.680)
**$CC_{1/2}$**	0.928 (0.349)	0.989 (0.354)	0.987 (0.284)	1.000 (0.417)
**Total observations**	357343 (35614)	311805 (30063)	223386 (22975)	580250 (42075)
**Unique observations**	36906 (3639)	34197 (3356)	21897 (2151)	59663 (5868)
**Space group**	P 31 2 1	P 31 2 1	P 31 2 1	P 31 2 1
**Unit cell dimensions (Å,Å,Å,°,°,°)**	89.541, 89.541, 106.212, 90, 90, 120	89.518, 89.518, 106.110, 90, 90, 120	89.566, 89.566, 106.360, 90, 90, 120	89.579, 89.579, 106.234, 90, 90, 120
**Solvent content (%)**	45.85	43.33	42.93	48.34
**R_{work} (%)**	16.95	18.20	16.23	16.65
**R_{free} (%)**	20.15	21.39	20.73	18.58
**RMS bonds (Å)**	0.013	0.013	0.013	0.011
**RMS angles (°)**	1.226	1.250	1.230	1.124
**Ramachandran outliers (%)**	0.36	0.35	0.35	0.35
**Ramachandran favored (%)**	96.80	96.45	96.10	97.16
**Clashscore**	1.06	2.33	2.13	2.08

Table S4: Crystallographic statistics.
Overall statistics given first (statistics for highest-resolution bin given in parentheses).

Table S5 is provided as an XLSX file.

Table S5: HDX-MS and crystallographic IADDAT difference values for all catalytic domain PTP1B variants.

Change in HDX rate (mutant-WT ∆%deuteration) and mean IADDAT value for HDX-reportable residues between mutant and wild-type for each peptide.
